# Modification of Sensory Expression of 3-Isobutyl-2-methoxypyrazine in Wines through Blending Technique

**DOI:** 10.3390/molecules26113172

**Published:** 2021-05-26

**Authors:** Mengqi Ling, Yu Zhou, Yibin Lan, Chifang Cheng, Guangfeng Wu, Changqing Duan, Ying Shi

**Affiliations:** 1 Center for Viticulture & Enology, College of Food Science and Nutritional Engineering, China Agricultural University, Beijing 100083, China; knighhtt@cau.edu.cn (M.L.); zhouyu@alu.cau.edu.cn (Y.Z.); lanyibin@cau.edu.cn (Y.L.); wumaple@cau.edu.cn (G.W.); chqduan@cau.edu.cn (C.D.); 2 Key Laboratory of Viticulture and Enology, Ministry of Agriculture and Rural Affairs, Beijing 100083, China; 3 Xinjiang CITIC Guoan Wine Co. Ltd., Manasi, Changji 832200, China; chengchifang@citicguoanwine.com

**Keywords:** 3-isobutyl-2-methoxypyrazines, volatile compounds, GC-MS, wine blending, bottle aging, sensory interaction, aroma modification

## Abstract

Sensory interactions exist between 3-alkyl-2-methoxypyrazines and various volatiles in wines. In this study, the binary blending of Cabernet Franc wines containing high levels of MPs and three monovarietal red wines with two proportions was conducted after fermentation. Volatiles were detected by gas chromatography-mass spectrometry (GC-MS), and wines were evaluated by quantitative descriptive analysis at three-month intervals during six-month bottle aging. Results showed blending wines exhibited lower intensity of ‘green pepper’, especially CFC samples blended by Cabernet Sauvignon wines with an even higher concentration of 3-isobutyl-2-methoxypyrazine (IBMP). Based on Pearson correlation analysis, acetates could promote the expression of ‘tropical fruity’ and suppress ‘green pepper’ caused by IBMP. Positive correlation was observed among ‘green pepper’, ‘herbaceous’, and ‘berry’. The concentration balance between IBMP and other volatiles associated with ‘green pepper’ and fruity notes was further investigated through sensory experiments in aroma reconstitution. Higher pleasant fruity perception was obtained with the concentration proportion of 1-hexanol (1000 μg/L), isoamyl acetate (550 μg/L), ethyl hexanoate (400 μg/L), and ethyl octanoate (900 μg/L) as in CFC samples. Blending wines with proper concentration of those volatiles would be efficient to weaken ‘green pepper’ and highlight fruity notes, which provided scientific theory on sensory modification of IBMP through blending technique.

## 1. Introduction

3-alkyl-2-methoxypyrazines (MPs) are a group of substances detected in wines made from Sauvignon Blanc, Cabernet Sauvignon, Cabernet Franc, Merlot, Carmenere, Semillon, and other varieties [1]. Those compounds contribute to the ‘green pepper’ of wine aroma and have extremely low thresholds, ranging from 2 to 16 ng/L detected in wines [2]. With the increasing sensitivity of detection technology, seven MPs have been identified in grapes and wines [3]. Among them, 3-isopropyl-2-methoxypyrazine (IPMP), 3-sec-butyl-2-methoxypyrazine (SBMP), and 3-isobutyl-2-methoxypyrazine (IBMP) are the most abundant in grapes and wines, especially IBMP with undesirable herbaceous, green, and vegetal notes. IPMP has been reported only a few cases of its concentration exceeding the detection threshold and impacting wine flavor, while SBMP exhibits limited effects on wine quality [4].

However, aroma characteristics of wines cannot be explained only from the knowledge of aroma composition alone. Interactions among various aromas play an important role in determining wine qualities. Although IBMP is considered to be responsible for ‘green pepper’ notes in wines [2,5], in some recent research, poor correlation was obtained when concentration of MPs was compared with aromatic intensities of ‘green pepper’ attributes, because of the contribution of other aromatic compounds such as varietal thiols, C6 alcohols, and dimethyl sulfide, and the suppressive effect of the reductive aromas or aldehydes on fresh green attribute should not be underestimated [6,7]. ‘Green’ aroma contributed by MPs was reported to be suppressed by the presence (or higher concentrations) of 4-ethylphenol, 4-ethyl guaiacol, and isopentanoic acid through CATA methodology analysis [8]. Previous studies also found the presence of volatile phenols may manifest ‘green’ characteristics such as ‘herbaceous’ and ‘grassiness’ even if IBMP levels are low [9,10]. In addition to ‘green pepper’, IBMP could contribute to other attributes along with some aroma-active compounds in wines, as it was found positively associated with ‘woody’ and ‘tobacco’ flavor in wines [11]. Although IBMP could be considered appropriate for some wine varieties by adding aroma complexity, it is generally regarded as negative traits, as the presence of IBMP might also lead to the indirect suppression effect of ‘clear/fruity’ notes in both white and red wines [12]. The masking effect of IBMP on the ‘tropical passionfruit’ notes contributed by 3-mercaptohexan-1-ol was investigated [13,14]. Therefore, viticultural and enological treatments have long been trialed to remove MPs and to reduce MP-derived greenness [12].

During winemaking, the blending technique after fermentation (‘*coupage*’) is conducted through blending wines with different characteristics to increase wine quality, mainly by equilibrating the composition of wines, increasing stability and complexity, and modulating wine flavor with specific purpose [15]. However, although chemical components usually follow linear relationships as proportional change, aroma perception in blending wines is more complex to be regulated because of sensory interactions among volatile compounds [16]. The aim of this work is to investigate interactions between IBMP and other volatiles and provide scientific theory on wine blending technique. Sensory expression of IBMP was modified through a binary blending design of one dry red wine that contained high levels of MPs with three different monovarietal red wines. Combining analysis of different blending wines at three-month intervals during six-month bottle aging with the results of reconstituted aroma matrices, the effect of green pepper aroma on wines and the contribution of other aroma active compounds was investigated.

## 2. Results and Discussion

### 2.1. Sensory and Chemical Profiles of Monovarietal Wines

**Figure 1:**
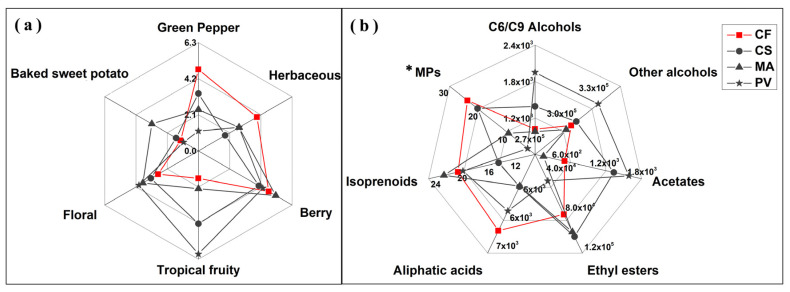
Sensory characteristics (**a**) and volatile profiles (**b**) of four monovarietal wines. CF, CS, MA, and PV refer to *Vitis vinifera* L. cv Cabernet Franc, Cabernet Sauvignon, Marselan, and Petit Verdot wines, respectively. The concentration of different compound categories in (**b**) is in units of μg/L except * MPs is in units of ng/L. Detailed information of volatiles is listed in Table S1.

The blending experiment design was based on different aroma characteristics of four monovarietal wines. According to sensory results in Figure 1a, CF wines (Cabernet Franc) exhibited high intensity of ‘green pepper’ and ‘herbaceous’. CS wines (Cabernet Sauvignon) also had relatively high intensity of ‘green pepper’, while having the lowest intensity of ‘herbaceous’. In MA wines (Marselan), the note of ‘baked sweet potato’ was identified as a combined description of ‘caramel’, ‘fragrance’, ‘sweet’, and ‘light toasted flavor’ by panelists, which might be accordant to ‘honey’ and ‘caramel’ descriptions obtained in MA wines in a previous study [17]. As for PV wines (Petit Verdot), the intensity of ‘green pepper’ was much lower than the other three monovarietal wines and the note of ‘tropical fruity’ had higher intensity, especially when compared to CF wines. The difference of intensities in ‘berry’ and ‘floral’ between four monovarietal wines was slight.

Aroma characteristic is regarded to be determined by volatile composition in wines. A total of 91 volatile compounds were detected in wines in this study, which were specifically divided into 7 C6/C9 alcohols, 17 other alcohols, 6 acetates, 18 ethyl esters, 17 other esters, 6 aliphatic acids, 8 isoprenoids, 9 other compounds, and 3 MPs. Detailed information and compound numbers are shown in Table S1. A radar map of the total concentration of compounds in each category of wine aroma was conducted (Figure 1b). CF wines exhibited lower concentration level of C6/C9 alcohols and acetates, especially comparing to PV wines. In addition to C6/C9 alcohols and acetates, the concentration of other alcohols was highest in PV wines, while the level of ethyl esters and MPs was lowest. Isoamyl acetate (banana and sweet notes) and phenethyl acetate (rose, honey, and fruity notes) are regarded as important acetates in wines [6,18], which could contribute to high intensity of ‘tropical fruity’ in PV wines. On the contrary, MA wines had the lowest concentration of acetates and higher level of isoprenoids and ethyl esters. As for CS wines, the main trait was that C6/C9 alcohols, MPs, ethyl esters and acetates all remained at relatively high levels, representing a more abundant volatile profile.

The distinction in the sensory score of ‘green pepper’ in monovarietal wines (Figure 1a) was consistent with the difference of MPs (Figure 1b). The high levels of MPs could be the reason for the highest score in ‘green pepper’ and ‘herbaceous’ in CF wines. Among three MPs detected in this work, IBMP concentration was higher, compared to IPMP and SBMP (Table S1), and it is regarded as the most important compound contributing to the notes of ‘green bell pepper’, ‘leafy’, and ‘herbaceous’ [19]. As a result of the sensory characteristics and high concentration of MPs, CF wines were regarded as base wines, and the other three monovarietal wines were treated as counterpart wines to participate in binary blending. Since a difference in volatiles such as C6/C9 alcohols, acetates, and ethyl esters existed among CS, MA, and PV wines, the influence of these volatiles on modifying the sensory expression of MPs was further investigated through the experiments of blending.

### 2.2. Impact of Blending Types and Proportions on Chemical and Sensory Profiles of Blending Wines

**Figure 2:**
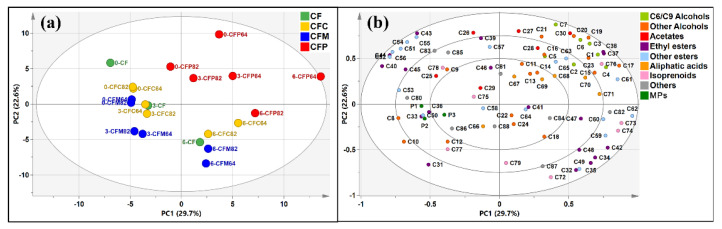
Principal component analysis of volatile compounds in CF wines and blending samples. (**a**) Score plot for wine sample differentiation at three-month intervals during six-month bottle aging. CFC, CFM, and CFP refer to binary blending samples of CF wines × CS wines, CF wines × MA wines, and CF wines × PV wines, respectively. The numbers 82 and 64 refer to CF wines accounted for 80% and 60%. The front number represents the length of aging (0, 3, and 6 months); (**b**) Scattering loading biplot of the coordinates of volatile compounds. Numbers of compounds are listed in Table S1.

Volatile chemical and sensory profiles in monovarietal CF wines and three binary blending wines (CS, MA, and PV wines) with two proportions were analyzed at three-month intervals during six-month bottle aging. The binary blending samples are denominated as CFC, CFM, and CFP. The abbreviation of ‘82’ refers to CF base wines blended in proportion of 80% with the other wines blended in a proportion of 20%, while ‘64’ refers to CF base wines blended in proportion of 60% with the other wines blended in proportion of 40%. Based on the PCA result (Figure 2a), ‘82’ samples are located more closely to CF wine than ‘64’ samples, indicating that the volatile profile had great correlation with proportions. In addition, the distribution of ‘82’ and ‘64’ samples became more dispersed during bottle aging, especially in the binary blending samples CFC (CF wines × CS wines) and CFM (CF wines × MA wines), namely, CFC82 vs. CFC64 and CFM82 vs. CFM64. In other words, the blending procedure could not only change the concentration of chemicals but also influenced the evolution of volatiles during aging to lead to more differentiated volatile profiles between different blending proportions after six-month bottle aging. However, compared to the factor of blending proportion, the blending type was a more prominent factor resulting in greater difference. To be more specific, CFP samples (CF wines × PV wines) were mainly located in the first quadrant where PC1 and PC2 were both positive, while CF wines and the other two blending types could not be separated by quadrants, suggesting that the volatile profile in CFP samples differed more from CF wines than the other two blending types.

According to the scattering loading biplot (Figure 2b), three MPs were located in the inner ellipse in biplot of PCA, suggesting that the level of MPs was not the main factor that determined the difference between CF wines and blending samples. As for volatile compounds selected in the outermost ellipse to determine the PCA model, C6/C9 alcohols were located in the first quadrant, representing higher concentrations in CFP samples, which was accordant with the characteristic of PV counterpart wines (Figure 1b). Acetates were distributed in the quadrants where PC2 was positive, presenting at higher concentrations in samples of 0 M and CFP blending type. The distributions of ethyl esters and other esters were mainly gathered in the second and fourth quadrants, exhibiting higher concentrations in samples of CFC and CFM blending types. In other words, the regulation of volatile profile in blending wines was mostly dependent on characteristics of different counterpart wines and the differences between blending types could be maintained after six-month bottle aging.

**Figure 3:**
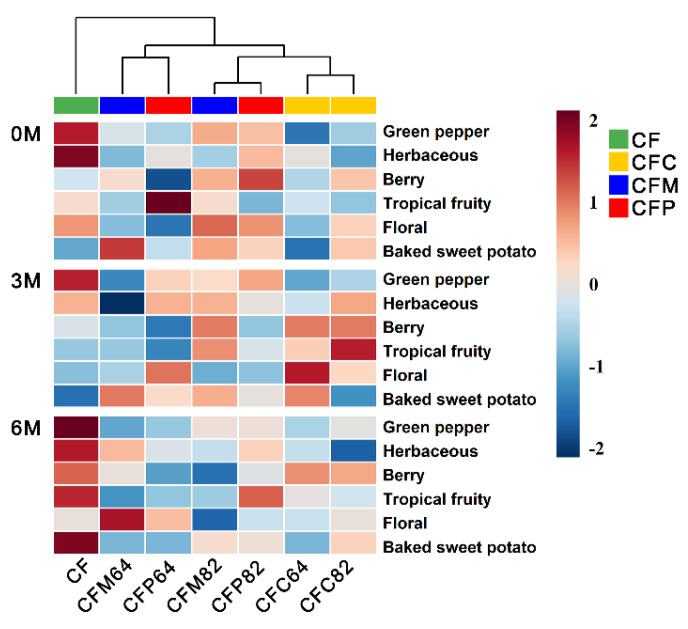
Clustering analysis of sensory scores in CF wines and blending samples.

**Figure 4:**
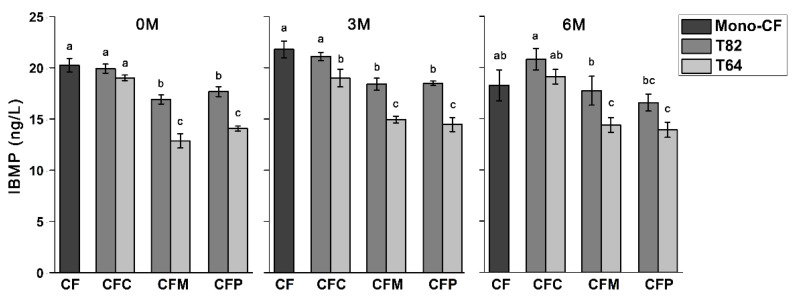
Difference of IBMP between CF wines and blending samples during bottle aging. Error bars are standard deviations. Different letters on error bars represent significant difference in each sampling point from Tukey’s HSD test (*p* < 0.05). T82 and T64 refer to CF wines accounted for 80% and 60%.

However, the sensory differences between blending types had some variation against the corresponding counterpart wines, which were visualized by clustered heatmap every three-month interval after bottling through sensory experiments (Figure 3). According to the results of quantitative descriptive analysis, the intensities in ‘green pepper’ and ‘herbaceous’ notes diminished in all binary blending samples comparing to CF base wines. In the blending types of CFM and CFP, CFM82 and CFP82 samples had higher intensities in ‘green pepper’ notes than CFM64 and CFP64 samples, which was in accordance with the concentration of IBMP (Figure 4). Although CFC samples had higher concentration of IBMP than CFM and CFP samples, CFC samples could be mostly distinguished from CF base wines with lower intensity of ‘green pepper’ and ‘herbaceous’ aromas. In addition, CFP samples did not exhibit prominent high intensity of ‘tropical fruity’, which was not consistent with sensory profile in PV counterpart wines that had the highest level of ‘tropical fruity’ among four monovarietal red wines (Figure 1a). The evolution of IBMP was also limited during six-month bottle aging (Figure 4). Therefore, the sensory expression of IBMP could be affected by other volatiles, and sensory interaction existed between the perception of ‘green pepper’ and other aroma description. It was evidenced that the sensory expression of MPs could be masked through interactions with other odorant compounds [20] and there existed a balance between fruitiness and greenness in wines according to a previous study [21]. However, wine aroma was influenced by a mixture of volatile compounds and could not be explained only through interaction between two individual compounds. The ‘green pepper’ notes in wines might be influenced by the modification of IBMP expression through the change of concentration proportion of volatile compounds in different blending types.

### 2.3. Correlation Analysis

Pearson correlation analysis was conducted to investigate the sensory interactions between MPs and other volatiles using four monovarietal wines and three binary blending wines (Table 1). Based on the relationship between aroma descriptions, only ‘tropical fruity’ showed a negative correlation with ‘green pepper’ (r = −0.38). The positive correlation between ‘green pepper’, ‘herbaceous’, and ‘berry’ indicated that a certain degree of fresh note caused by ‘green pepper’ and ‘herbaceous’ might promote the perception of ‘berry’ and vice versa. However, ‘berry’ was also positively correlated with ‘floral’ (r = 0.39), and ‘floral’ was positively related to ‘tropical fruity’ (r = 0.68). The complicated correlation between different aroma descriptions represented that one sensory note could be linked to other notes simultaneously. Expression of the ‘green pepper’ note was not only influenced by volatiles with direct correlation; compounds that correlated to other aroma descriptions, such as ‘tropical fruity’, ‘berry’, and ‘herbaceous’, could also influence the perception of ‘green pepper’.

As the results showed in Table 1, a total of 51 volatile compounds showed high correlation coefficients with sensory description and significant difference in proportion and blending type based on the results of two-way ANOVA in blending samples (*p* < 0.05). IBMP was positively correlated with the note of ‘green pepper’ as expected, which was in accordance with the theory that IBMP is described as pyrazic and musty-earthy, and it is known to be involved in the characteristic ‘green note’ of Cabernet-type wines [22]. However, the correlation coefficient between IBMP and ‘green pepper’ was not high (r = 0.49), and both IPMP and SBMP had poor correlation with ‘green pepper’ (*p* > 0.05). More studies have been focused on IBMP than on IPMP, and there is a lack of reports on SBMP affecting wine quality [4]. In this study, SBMP was positively correlated with ‘baked sweet potato’, indicating that it might contribute to some ‘caramel’ and ‘aldehydic’ flavor similarly to the note of IPMP reported in previous studies [22]. The weak correlation between MPs concentration and intensity of ‘green pepper’ attribute was also obtained in Sauvignon Blanc wines because of the contribution of other aromatic compounds such as varietal thiols, C6 alcohols, and dimethyl sulfide [7].

In addition to IBMP, 19 compounds showed correlation with ‘green pepper’. The correlation coefficients of C6/C9 alcohols with ‘green pepper’ was negative, which was different from the expectation that C6 alcohols could result in ‘basil’ and ‘cut grass’ notes and contribute to the perception of ‘green pepper’ in wines [5,23]. Meanwhile, the C6/C9 compounds detected in our research showed a positive correlation with ‘tropical fruity’ and ‘floral’ notes, while the negative correlation of C6 compounds with fruitiness found through partial least-squares regression (PLSR) in a previous study [21]. Among other alcohols, phenylethyl alcohol, which is important to ‘rose, honey’ as reported [24], was found negative correlated with ‘green pepper’ (r = −0.41) and was not significantly correlated with other notes. Four acetates were positively related to the note of ‘tropical fruity’ and showed negative correlation with ‘green pepper’ and ‘berry’. Ethyl esters had different contribution to wine aroma description. Ethyl hexanoate was positive to ‘green pepper’ (r = 0.44), while it was negative to ‘tropical fruity’ (r = −0.47) and ‘floral’ (r = −0.51), which was the same as IBMP. However, ethyl *(E)*-3-hexenoate and ethyl 2-hexenoate both showed a negative correlation to ‘green pepper’ (r = −0.43, r = −0.49, respectively). Although some compounds were not correlated with ‘green pepper’, their impact on the expression of IBMP could not be underestimated. For example, ethyl octanoate was negatively correlated with ‘berry’ (r = −0.40), ‘tropical fruity’ (r = −0.53), and ‘floral’ (r = −0.68) notes. A higher intensity of ‘berry’ note might lead to higher perception of ‘green pepper’ (r = 0.42), while a higher intensity of ‘tropical fruity’ note might suppress the perception of ‘green pepper’ (r = −0.38). Since there existed different correlation among different aroma descriptions (Table 1), the contribution of ethyl octanoate to ‘green pepper’ was more complicated.

Combining correlation analysis (Table 1) with sensory difference in different blending types (Figure 3), interactions between aroma compounds in wines was investigated. Although CFP samples had high levels of acetates and a relatively low level of IBMP, the fruity notes in CFP samples were lower and the scores in ‘green pepper’ and ‘herbaceous’ were higher than in CFC samples. The results indicated that the correlation between wine aroma with single volatiles was not enough to explain the aroma expression. The sensory characteristics of CFP samples were possibly due to the high levels of C6/C9 alcohols in CFP samples. Many researchers found that the excessive concentration of C6/C9 alcohols would inevitably contribute to herbaceous notes and suppress the expression of fruity notes [5,23]. On the contrary, CFC samples had the lowest sensory score of ‘green pepper’ even with the highest concentration of IBMP among three blending types (Figure 4). This might be due to the appropriate concentration proportion of other volatiles in CFC samples. In other words, CFC samples had ethyl esters with the highest concentration and C6/C9 alcohols and acetates with the second highest concentration. The presence of MPs at certain concentrations could also contribute to the complexity and typicity of some wines [12]. The proper balance between fruitiness and greenness was reported to be important to wine aroma quality [21], and the enhancing and masking effects between different volatile compounds are receiving more and more attention [6,25].

### 2.4. Interactions among IBMP, 1-Hexanol, Isoamyl Acetate, Ethyl Hexanoate, and Ethyl Octanoate

To confirm the importance of the balance between fruitiness and greenness (‘green pepper’ note) in wines, sensory differences in reconstituted aroma matrices of three levels of IBMP and three blending types of key volatile compounds were evaluated. 1-Hexanol, isoamyl acetate, ethyl hexanoate, and ethyl octanoate are compounds with high odor activity values (OAVs) in wines, of which the perception thresholds are 1100, 160, 80, and 580 μg/L, respectively [24]. These four compounds were also chosen because of high Pearson correlation coefficients with three sensory descriptions and significant differences in three blending types (Table 1). The addition concentration of standard volatiles in reconstituted aroma matrices were according to the levels of blending samples. The detailed design is described in Table 2.

**Figure 5:**
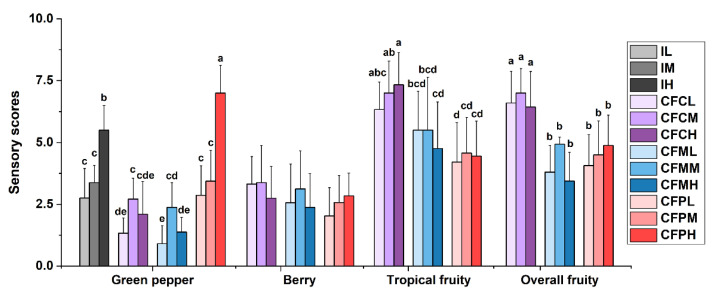
Sensory difference in reconstituted aroma matrices of three levels of IBMP and three blending types of key volatile compounds. Error bars are standard deviations. Different letters on error bars represent significant difference in each aroma description from Duncan’s multiple range test (*p* < 0.05). Sample abbreviation is described in Table 2.

According to Figure 5, the intensity of ‘green pepper’ was in accordance with the concentration of IBMP in CFP reconstituted matrices, while it was higher at a medium level of IBMP concentration in CFC and CFM reconstituted matrices. Although the intensities of ‘berry’ and ‘overall fruity’ notes exhibited no significant difference between three levels of IBMP in each type of reconstituted matrix (*p* > 0.05), the same variation tendency as ‘green pepper’ was observed. This indicated the positive correlation between ‘green pepper’ and berry-like fruity. In CFP reconstituted matrices, the perception of ‘tropical fruity’ was lowest even with highest concentration of isoamyl acetate (Table 2). At a high level of IBMP, the aroma composition of CFP reconstituted matrices would enhance the perception of ‘green pepper’. It was possibly because the level of 1-hexanol in CFP wines (1200 μg/L) exceeded its threshold (1100 μg/L) [24], which might suppress ‘tropical fruity’ and stimulate the perception of ‘green pepper’. The aroma composition of CFC and CFM reconstituted matrices could both decrease the perception of ‘green pepper’ comparing to corresponding levels of IBMP solution (IL, IM, IH). The intensities of ‘berry’ and ‘overall fruity’ had a higher trend at medium level of IBMP in CFC and CFM reconstituted matrices, suggesting the concentration of IBMP between 15.36 (low) and 19.20 ng/L (medium) might have a synergy effect on fruity. The scores of fruity descriptions (‘tropical fruity’ and ‘overall fruity’) were significantly higher in CFC reconstituted matrices than CFM reconstituted matrices, providing an appropriate concentration proportion of 1-hexanol (1000 μg/L), isoamyl acetate (550 μg/L), ethyl hexanoate (400 μg/L), and ethyl octanoate (900 μg/L) as the concentration in CFC reconstituted matrices (Table 2).

## 3. Materials and Methods

### 3.1. Wine Samples

Monovarietal dry red wines, namely, *Vitis vinifera* L. cv Cabernet Franc (CF), Cabernet Sauvignon (CS), Marselan (MA), and Petit Verdot (PV) were made strictly in accordance with the local winemaking procedures at Xinjiang CITIC Guoan Winery (Xinjiang, China) in 2018. The detailed physicochemical parameters of wine samples are shown in Table S2. After malolactic fermentation, the binary blending design was conducted as described in Table 3. CF wines were regarded as base wines. CS, MA, and PV wines were selected as counterpart wines. The binary blending samples were denominated as CFC, CFM, and CFP. CF base wines were blended in proportion of 80% (T82) and 60% (T64) to investigate whether 20% counterpart wines and 40% counterpart wines would alter the characteristic of base wines. After blending, wines were bottled in 750 mL bottles sealed with corks and stored in a cellar, with an average temperature of 16 ± 1 °C and relative humidity of 65 ± 5%. Two bottles of wine samples of each monovarietal wines and blending wines were randomly collected at three-month intervals during six-month bottle aging and stored at −20 °C for further analysis. The following are available online in Supplementary Materials: Table S1: Volatile compounds identified in this work and their aroma parameters; Table S2: Detailed physicochemical parameters of monovarietal wine samples; Table S3: Validation parameters of the detection of three 3-alkyl-2-methoxypyrazines; Table S4: Aroma attributes and corresponding definitions used in QDA by panels

### 3.2. Reagents and Standards

GC grade IPMP, SBMP, and IBMP were purchased from Sigma-Aldrich (St. Louis, MO, USA). Analytical grade solvents, including NaCl, NaOH, and tartaric acid, were obtained from Macklin Biochemical Co., Ltd. (Shanghai, China). HPLC grade ethanol and dichloromethane were supplied by Honeywell (Morris Township, NJ, USA). Aroma reference standards for quantification were purchased from Sigma-Aldrich (St. Louis, MO, USA) (Table S1).

### 3.3. Detection of 3-Alkyl-2-Methoxypyrazines

#### 3.3.1. Headspace Solid-Phase Microextraction (HS-SPME) Procedure

Extraction of MPs was carried out according to the method described by a previous study with slight modifications [26]. Wine samples were adjusted to pH 7.0 by the addition of 5 mol/L NaOH solution and then diluted to 5.4% (*v*/*v*) alcohol with model solution (2 g/L glucose and 7 g/L tartaric acid without ethanol, pH 7.0). Then, 5 mL of diluted sample was placed in a 20 mL vial with 1.5 g NaCl capped with a PTFE–silicon septum. The equilibration was conducted at 38 °C for 10 min and then 2 cm DVB/CAR/PDMS 50/30 µm SPME fiber (Supelco, Bellefonte, PA., USA) was inserted into the headspace of vial for 65 min to extract MPs. The injection was accomplished by a CTC CombiPAL autosampler (CTC Analytics, Zwingen, Switzerland).

#### 3.3.2. GC-MS Conditions

An Agilent 7890 GC equipped with an Agilent 5975 MS (Agilent Technologies, Inc. CA, USA) was used for the detection of MPs. After extraction, injection was conducted in splitless mode with an 8-min thermal desorption at 250 °C. The separation was carried out on a HP-INNOWAX capillary column (60 m × 0.25 mm id, 0.25 μm film thickness, J&W Scientific, Folsom, CA, USA). The flow rate of carrier gas, helium, was 1 mL/min. The GC temperature program was as follows: holding at 50 °C for 1 min, increasing at a rate of 3.0 °C/min to 110 °C, and increasing at a rate of 1.5 °C/min to 131 °C. The after run temperature was set as 220 °C for 10 min. The MSD transfer line heater was set at 250 °C, and the temperature of ion source and quadrupole were 250 °C and 150 °C, respectively. The MSD was operated in electron ionization (EI) mode at 70 eV and selected ion monitoring (SIM) with the selected mass channels for IPMP (*m/z* 137, 152), SBMP (*m/z* 124, 138, 151), and IBMP (*m/z* 124, 151, 166), with a dwell time of 100 ms. The quantification ions for IPMP, SBMP, and IBMP were *m/z* 137, 124, and 124, respectively.

#### 3.3.3. Method Validation

Method validation was evaluated in a matrix of dearomatized Marselan dry red wines. The dearomatization was conducted by three steps. Firstly, wines were extracted by dichloromethane with a ratio of 5:1 with magnetic stirring for 30 min at room temperature. The extraction was repeated twice, and the organic phase, which contained most volatile aroma compounds, was discarded, while the remaining aqueous phase was retained. Secondly, the collected aqueous phase was distilled by SAFE distillation (Glasblaserei Bahr, Freising, Germany) coupled to a vacuum pump (KYKY Technology (Beijing) Co. Ltd., Beijing, China) to remove the remaining volatiles [27]. Thirdly, the residue was mixed with ethanol and pure water to reach 13% alcohol concentration and original volume of wine samples, and it was adjusted to pH 3.5. The calibration solutions were prepared at seven concentration levels. The limits of detection (LODs) and quantification (LOQs) were calculated by MSD ChemStation Data Analysis (Agilent Technologies, Inc. CA, USA) for the typical signal-to-noise (S/N) ratios of 3 and 10, respectively. Red wines spiked with three concentration levels of MPs, approximately 5, 15, and 30 ng/L, were conducted to evaluated recovery (%). Red wines spiked with 15 ng/L of MPs were conducted to evaluated the relative standard deviation (RSD, %), including the repeatability (intraday precision, five replicates of the same sample within a day) and the reproducibility (interday precision, five replicates of the same sample over a period of five days). Extraction and analysis were conducted in triplicate. The validation results containing the accuracy of the detection method of MPs are listed in Table S3.

### 3.4. Analysis of Volatile Compounds

Volatile compounds in wines were extracted with 2 cm DVB/CAR/PDMS 50/30 µm SPME fiber and then analyzed using an Agilent 7890 GC equipped with an Agilent 5975 MS [28]. Briefly, a 5 mL wine sample was placed in a 20 mL vial with the addition of 1.5 g NaCl and 10 μL 4-methyl-2-pentanol (internal standard, 1 g/L). After 30 min of equilibration at 40 °C, the fiber was injected into the headspace of the vial staying 30 min and then desorbed in a GC injector for 8 min at 250 °C with a split ratio of 4:1. A HP-INNOWAX capillary column was used and the temperature program was conducted as follows: holding 50 °C for 1 min, increasing at a rate of 3.0 °C/min to 220 °C, and holding 220 °C for 5 min. Volatile compounds were identified by comparing the obtained mass spectra and retention indices (RI) with those of aroma reference standard and compounds in the NIST 11 MS database. Standard volatile compounds were dissolved with ethanol (HPLC grade) and diluted into twelve levels in the synthetic matrix (13% ethanol with 2 g/L glucose and 7 g/L tartaric acid, pH 3.5) and analyzed using the same method as wine samples. Quantitation was conducted through calibration curves of aroma reference standards. Analysis of wine samples was performed in duplicate.

### 3.5. Sensory Analysis

#### 3.5.1. Panel Training

Sensory panel members were selected from faculties, staff, and students from the Center for Viticulture & Enology, China Agricultural University. After successive training sessions involving recognition, sorting, and intensity rating, panel assessment was tested according to the method described in a previous study [27]. A total of 16 trained panelists (five males, 11 females, 20–29 years old) participated in the sensory experiment. The panel agreed on six aroma attributes, including ‘green pepper’, ‘herbaceous’, ‘berry’, ‘tropical fruity’, ‘floral’, and ‘baked sweet potato’. The training references of each aroma attribute were prepared by aroma standards from Sigma-Aldrich (St. Louis, MO, USA), food essence from Aipu Food Industry (Shanghai, China), and natural products (Table S4).

#### 3.5.2. Quantitative Descriptive Analysis (QDA)

Panelists were asked to evaluate the intensity of six aroma attributes described above on a 11-point scale (0 = very low intensity, 10 = strong intensity). The formal sessions were carried out every three months with the same panel. Before each formal session, a CF monovarietal dry red wine was used to recall the standard of scoring and a consensus was reached by the panel. During sensory testing, all wine samples were provided randomly to eliminate the system error of sample order. The outlier values of sensory data were eliminated by conducting boxplot analysis before further statistical analysis [29].

#### 3.5.3. Sensory Verification of the Interaction among IBMP, 1-Hexanol, Isoamyl Acetate, Ethyl Hexanoate, and Ethyl Octanoate

The synthetic matrix (13% ethanol with 2 g/L glucose and 7 g/L tartaric acid, pH 3.5) was used as the model wine solution. Three levels of IBMP (low concentration, IL; medium concentration, IM; high concentration, IH), together with one blank sample (the model wine solution) were presented in a random order to evaluate the intensity of ‘green pepper’. The addition concentration of 1-hexanol, isoamyl acetate, ethyl hexanoate, and ethyl octanoate in reconstituted matrices was determined by the concentration in corresponding blending samples, CFC, CFM, and CFP (Table 1). Three types of reconstituted matrices were spiked with three levels of IBMP, which were described as CFCL, CFCM, CFCH, CFML, CFMM, CFMH, CFPL, CFPM, and CFPH (Table 2). The intensity of ‘green pepper’, ‘berry’, ‘tropical fruity’, and ‘overall fruity’ were scored.

### 3.6. Statistical Analysis

One-way and two-way analysis of variance (ANOVA) test and Pearson correlation analysis were conducted with SPSS20.0 software (SPSS, Chicago, IL, USA). The clustered heatmap was carried out by ‘pheatmap’ package in R environment (3.4.0) (http://www.r-project.org/, accessed on 21 April 2017), and graphs were prepared by ‘ggplot2’ package in R. Principal component analysis (PCA) was performed by SIMCA 14.1 (Umetrics, Malmö, Sweden). The rest of the graphs were drawn using OriginPro 9.1 (OriginLab, Northampton, MA, USA).

## 4. Conclusions

Binary blending after fermentation of one base wine (CF) with high levels of MPs and three different counterpart wines (CS, MA, and PV) with two proportions (CF accounted for 80% and 60%) was conducted. Results showed the regulation of volatile profile in blending wines mostly depended on characteristics of different counterpart wines and the influence of blending type was more prominent than proportion. However, although PV counterpart wines had the highest intensity of ‘tropical fruity’ and lowest intensity of ‘green pepper’, the suppression effect of ‘green pepper’ in CFP blending samples was not the most effective. Among three blending types, it was CFC blending samples that differed most from CF wines in sensory scores because of low perception in ‘green pepper’ even with the highest concentration of IBMP.

Therefore, reducing the concentration of MPs through the blending technique with wines of low MPs levels was not sufficient to decrease the note of ‘green pepper’. ‘Green pepper’ was not only influenced by volatiles with direct correlation; compounds that correlated to other aroma descriptions could also have affected the perception of ‘green pepper’. Volatiles contributing to ‘tropical fruity’ might suppress the perception of ‘green pepper’. Positive correlation was observed among ‘green pepper’, ‘herbaceous’, and ‘berry’, indicating that the concentration balance of volatiles associated with fruitiness and greenness is more important to wine aroma quality than the concentration of a single compound. Sensory experiments in reconstituted aroma matrices verified the concentration proportion of 1-hexanol (1000 μg/L), isoamyl acetate (550 μg/L), ethyl hexanoate (400 μg/L), and ethyl octanoate (900 μg/L), as the concentration in CFC reconstituted matrices was more efficient to weaken the prominent undesirable herbaceous aroma and highlight fruity notes. The impact of 1-hexanol, isoamyl acetate, ethyl hexanoate, and ethyl octanoate on the expression of IBMP would provide scientific guidance on the blending technique. Our study investigated the combined influence of four key volatiles on IBMP due to the difference of blending wine samples. Further investigation could focus on mutual effects of aroma compounds on the sensory expression of IBMP at the molecular level.

## Figures and Tables

**Table 1:** Volatile compounds with high Pearson correlation coefficients with sensory descriptions and two-way ANOVA results for proportion and binary blending type factors and their interaction (proportion × blending) effect.

NO. ^1^	Compounds	Pearson Correlation Coefficient ^2^						Two-Way ANOVA ^3^					
		Green Pepper	Herbaceous	Berry	Tropical Fruity	Floral	Baked Sweet Potato	Proportion		Binary Blending Type			Proportion × Blending
								T82	T64	CFC	CFM	CFP	
*Sensory scores*													
Green Pepper		1.00						3.32 ^b^	3.82 ^a^ *	ns.			ns.
Herbal		0.41	1.00					ns.		ns.			ns.
Berry		0.42	0.59	1.00				ns.		ns.			ns.
Tropical fruity		−0.38	nc.	nc.	1.00			ns.		ns.			ns.
Floral		nc.	nc.	0.39	0.68	1.00		ns.		ns.			ns.
Baked sweet potato		nc.	nc.	nc.	nc.	nc.	1.00	ns.		ns.			ns.
*C6/C9 alcohols*													
C1	1-Hexanol	−0.55	nc.	nc.	0.73	0.52	nc.	960.54 ^b^	1039.05 ^a^ **	1006.19 ^b^	879.23 ^c^	1113.98 ^a^ **	**
C2	*(E)*-3-Hexen-1-ol	−0.56	nc.	nc.	0.75	0.58	nc.	36.18 ^b^	38.93 ^a^ **	38.57 ^a^	34.39 ^b^	39.70 ^a^ **	**
C3	*(Z)*-3-Hexen-1-ol	nc.	nc.	nc.	0.56	0.46	nc.	50.06 ^b^	55.53 ^a^ **	39.92 ^b^	42.02 ^b^	76.44 ^a^ **	**
C5	*(Z)*-2-Hexen-1-ol	nc.	nc.	nc.	0.42	nc.	nc.	ns.		12.15 ^b^	11.27^b^	14.38^a^ **	ns.
C6	2-Nonanol	−0.49	nc.	nc.	0.55	0.41	nc.	ns.		1.51 ^b^	1.32 ^c^	1.95 ^a^ **	ns.
C7	1-Nonanol	−0.47	nc.	−0.43	0.52	nc.	nc.	1.94 ^b^	2.11 ^a^ **	1.94 ^b^	1.81 ^c^	2.33 ^a^ **	**
*Other alcohols*													
C12	1-Pentanol	0.64	nc.	nc.	−0.70	−0.49	nc.	72.60 ^a^ **	68.96 ^b^	ns.			ns.
C15	1-Octen-3-ol	nc.	nc.	nc.	0.58	0.58	nc.	ns.		2.50 ^b^	2.26 ^c^	2.67 ^a^ **	ns.
C16	1-Heptanol	−0.51	nc.	nc.	0.59	nc.	nc.	6.44 ^b^	7.03 ^a^ **	7.55 ^a^ **	5.44 ^b^	7.22 ^a^	**
C17	2-Heptanol	−0.50	nc.	nc.	0.70	0.59	nc.	4.04 ^b^	4.34 ^a^ **	3.84 ^b^	3.81 ^b^	4.92 ^a^ **	**
C18	2-Ethylhexanol	nc.	0.42	nc.	nc.	nc.	nc.	ns.		2.38 ^a^ **	1.79 ^b^	1.83 ^b^	ns.
C20	1-Decanol	nc.	nc.	nc.	0.49	nc.	nc.	2.69 ^b^	2.77 ^a^ **	2.64 ^b^	2.62 ^b^	2.92 ^a^ **	**
C21	Methionol	nc.	nc.	−0.40	nc.	nc.	nc.	ns.		1009.63 ^b^	1066.76 ^b^	1223.74 ^a^ **	ns.
C23	Phenylethyl alcohol	−0.41	nc.	nc.	nc.	nc.	nc.	20321.71 ^b^	24647.00 ^a^ **	19968.04 ^b^	20741.73 ^b^	26743.30 ^a^ **	ns.
C24	1-Dodecanol	nc.	0.41	nc.	nc.	nc.	nc.	2.19 ^b^	2.23 ^a^ *	ns.			ns.
*Acetates*													
C26	Isobutyl acetate	−0.41	nc.	−0.51	0.50	nc.	nc.	ns.		50.26 ^a^ **	40.28 ^b^	49.30 ^a^	*
C27	Isoamyl acetate	−0.50	nc.	−0.49	0.60	nc.	nc.	441.97 ^a^	506.90 ^a^ **	546.69 ^a^	292.93 ^b^	583.67 ^a^ **	**
C28	Hexyl acetate	−0.58	nc.	−0.41	0.61	nc.	nc.	1.59 ^b^	1.93 ^a^ **	2.04 ^b^	0.94 ^c^	2.30 ^a^ **	**
C30	Phenethyl acetate	−0.56	nc.	−0.42	0.65	nc.	nc.	15.61 ^b^	19.12 ^a^ **	14.51 ^b^	13.14 ^b^	24.45 ^a^ **	**
*Ethyl esters*													
C36	Ethyl hexanoate	0.44	nc.	nc.	−0.47	−0.51	nc.	373.98 ^a^ **	346.74 ^b^	408.86 ^a^ **	346.91 ^b^	325.30 ^c^	**
C37	Ethyl *(E)*-3-hexenoate	−0.43	nc.	nc.	0.70	0.56	nc.	0.52 ^b^	0.60 ^a^ **	0.47 ^b^	0.37 ^c^	0.83 ^a^ **	**
C38	Ethyl 2-hexenoate	−0.49	nc.	nc.	0.74	0.55	nc.	1.53 ^b^	1.80 ^a^ **	1.57 ^b^	1.18 ^c^	2.25 ^a^ **	**
C39	Ethyl heptanoate	nc.	nc.	−0.55	0.42	nc.	nc.	0.74 ^b^	0.77 ^a^ **	0.78 ^a^	0.70 ^b^	0.78 ^a^ **	ns.
C40	Ethyl octanoate	nc.	nc.	−0.40	−0.53	−0.68	nc.	ns.		873.39 ^a^ **	792.59 ^b^	741.61 ^b^	ns.
C45	Ethyl 9-decenoate	nc.	−0.40	nc.	nc.	nc.	nc.	ns.		5.09 ^a^ **	2.51 ^b^	2.49 ^b^	**
C46	Ethyl dodecanoate	nc.	nc.	nc.	−0.44	−0.55	nc.	ns.		33.66 ^a^ *	29.09 ^b^	32.90 ^a^	ns.
C47	Ethyl tetradecanoate	nc.	0.43	nc.	nc.	nc.	nc.	ns.		59.53 ^a^ **	42.04 ^b^	55.13 ^a^	ns.
C48	Ethyl hexadecanoate	nc.	0.49	0.51	nc.	nc.	nc.	ns.		172.81 ^a^ **	121.47 ^b^	126.52 ^b^	ns.
*Other esters*													
C50	Methyl hexanoate	0.54	nc.	nc.	−0.53	−0.61	nc.	1.50 ^a^ **	1.38 ^b^	1.58 ^a^ **	1.40 ^b^	1.34 ^b^	**
C52	Methyl octanoate	nc.	nc.	−0.45	−0.56	−0.70	nc.	ns.		2.76 ^a^ *	2.52 ^b^	2.50 ^b^	ns.
C57	Isopentyl decanoate	nc.	nc.	−0.51	nc.	−0.49	nc.	ns.		3.48 ^a^	3.30 ^b^	3.51 ^a^ **	ns.
C59	Isoamyl lactate	nc.	0.56	nc.	nc.	nc.	nc.	ns.		190.51 ^b^	224.56 ^a^	232.08 ^a^ **	ns.
C60	Diethyl succinate	nc.	nc.	nc.	0.47	0.43	−0.42	2412.34 ^b^	3503.38 ^a^ **	4089.48 ^a^ **	1500.02 ^b^	3284.08 ^a^	ns.
C61	Ethyl benzoate	−0.55	nc.	nc.	0.77	0.62	nc.	1.80 ^b^	1.89 ^a^ **	1.85 ^b^	1.76 ^c^	1.93 ^a^ **	**
C62	Ethyl phenylacetate	nc.	nc.	nc.	0.52	0.60	nc.	ns.		2.03 ^b^	2.07 ^b^	2.27 ^a^ **	ns.
*Aliphatic acids*													
C70	Octanoic acid	nc.	nc.	nc.	nc.	nc.	−0.53	ns.		1673.20 ^a^	1428.26 ^b^	1756.35 ^a^ **	ns.
C71	n-Decanoic acid	nc.	0.49	nc.	nc.	nc.	−0.51	ns.		204.36 ^b^	184.89 ^c^	217.41 ^a^ **	ns.
*Isoprenoids*													
C72	*p*-Cymene	nc.	0.50	0.51	nc.	nc.	nc.	ns.		3.32 ^b^	5.24 ^a^ **	3.30 ^b^	ns.
C73	Linalool	nc.	nc.	nc.	0.54	0.63	nc.	ns.		2.29 ^c^	2.54 ^b^	2.85 ^a^ **	ns.
C74	*α*-Terpineol	nc.	0.52	nc.	nc.	0.47	nc.	1.37 ^b^	1.53 ^a^ **	1.31 ^b^	1.47 ^a^	1.56 ^a^ **	ns.
C75	Citronellol	nc.	nc.	nc.	nc.	nc.	0.57	3.79 ^b^	4.39 ^a^ **	3.50 ^c^	4.76 ^a^ **	4.03 ^b^	ns.
C76	*(6E)*-Nerolidol	nc.	nc.	nc.	0.56	0.44	−0.46	3.45 ^b^	3.56 ^a^ **	3.46 ^b^	3.36 ^c^	3.69 ^a^ **	**
C77	Theaspirane	nc.	nc.	nc.	nc.	nc.	0.61	0.42 ^b^	0.43 ^a^ **	0.41 ^b^	0.46 ^a^ **	0.41 ^b^	**
C79	*β*-Ionone	nc.	0.51	0.63	nc.	nc.	nc.	ns.		1.98 ^a^ **	1.90 ^a^	1.64 ^b^	ns.
*Others*													
C80	Styrene	nc.	nc.	nc.	nc.	nc.	0.70	ns.		1.13 ^b^	1.54 ^a^ **	1.15 ^b^	*
C81	Benzaldehyde	−0.57	−0.52	−0.51	nc.	nc.	nc.	3.41 ^b^	3.55 ^a^ **	ns.			ns.
C82	Phenylacetaldehyde	−0.47	nc.	nc.	nc.	nc.	nc.	9.60 ^b^	10.24 ^a^ **	ns.			ns.
C86	Furfural	nc.	nc.	0.53	nc.	nc.	nc.	ns.		43.01 ^ab^	44.94 ^a^ *	33.76 ^b^	ns.
C87	Ethyl 2-furoate	nc.	0.50	0.51	nc.	nc.	nc.	ns.		2.68 ^a^ **	2.52 ^a^	2.33 ^b^	ns.
*3-Alkyl-2-methoxypyrazines*													
P2	SBMP (ng/L)	nc.	nc.	nc.	nc.	nc.	0.56	ns.		2.27 ^b^	2.83 ^a^ **	2.15 ^b^	ns.
P3	IBMP (ng/L)	0.49	nc.	nc.	−0.56	−0.60	nc.	18.62 ^a^ **	15.75 ^b^	19.82 ^a^ **	15.88 ^b^	15.87 ^b^	**

^1^ Detailed information of volatile compounds are listed in Table S1. ^2^ “nc.” indicates that the correlation was not significant (*p* > 0.05). ^3^ Mean values with different letters represent significant difference in the factor of proportion and the factor of binary blending type, respectively; “ns.”: *p* > 0.05; “*”: *p* < 0.05; “**”: *p* < 0.01; The concentration of volatile compounds is in units of μg/L except SBMP and IBMP.

**Table 2:** Synthetic matrix design of three blending types and three level of IBMP for sensory verification.

Synthetic Matrix	Concentration in Aroma Reconstitution (μg/L)				IBMP (ng/L)		
	1-Hexanol	Isoamyl Acetate	Ethyl Hexanoate	Ethyl Octanoate	Low	Medium	High
IL	—	—	—	—	15.36	—	—
IM					—	19.20	—
IH					—	—	23.04
CFCL	1003.62	551.81	402.02	896.78	15.36	—	—
CFCM					—	19.20	—
CFCH					—	—	23.04
CFML	880.78	298.90	350.24	791.28	15.36	—	—
CFMM					—	19.20	—
CFMH					—	—	23.04
CFPL	1202.26	586.30	328.92	738.53	15.36	—	—
CFPM					—	19.20	—
CFPH					—	—	23.04

**Table 3:** Binary blending design of wine samples.

Blending Wine Samples	Monovarietal Wines		Binary Blending	Proportion ^1^
	Base Wine	Counterpart Wines		
CFC82	CF	CS	CSC	T82
CFC64				T64
CFM82		MA	CSM	T82
CFM64				T64
CFP82		PV	CSP	T82
CFP64				T64

^1^ T64: 60% CF + 40% corresponding counterpart wines; T82: 80% CF + 20% corresponding counterpart wines. The proportion was based on volume.

## Data Availability

The data presented in this study are available in the supplementary material.

## Supplementary Materials

The following are available online. Table S1: Volatile compounds identified in this work and their aroma parameters; Table S2: Detailed physicochemical parameters of monovarietal wine samples; Table S3: Validation parameters of the detection of three 3-alkyl-2-methoxypyrazines; Table S4: Aroma attributes and corresponding definitions used in QDA by panels.

## Sample Availability

Samples of the compounds are not available from the authors.
